# WASF3 overexpression affects the expression of circular RNA hsa-circ-0100153, which promotes breast cancer progression by sponging hsa-miR-31, hsa-miR-767-3p, and hsa-miR-935

**DOI:** 10.1016/j.heliyon.2023.e22874

**Published:** 2023-11-29

**Authors:** Majid Mokhtari, Samane Khoshbakht, Mohammad Esmaeil Akbari, Moravveji Sayyed Sajjad

**Affiliations:** a Department of Bioinformatics, Kish International Campus, University of Tehran, Kish Island, Iran; b Cancer Research Center, Shahid Beheshti University of Medical Sciences, Tehran, Iran

**Keywords:** Cancer, Oncology, Gene expression, circRNA, miRNA, Bioinformatics

## Abstract

**Background:**

The *WASF3* gene has been linked to promoting metastasis in breast cancer (BC) cells, and low expression reduces invasion potential. Circular RNAs (circRNAs) function as microRNA (miRNA) modulators and are involved in cancer progression, but the relationship between these factors remains unclear.

**Methods:**

This study used bioinformatics methods and a computational approach to investigate the role of circRNAs and miRNAs in the context of *WASF3* overexpression. Differentially expressed mRNAs, circRNAs, and miRNAs were identified using Gene Expression Omnibus (GEO) datasets. A competing endogenous RNA (ceRNA) network was constructed based on circRNA-miRNA pairs and miRNA-mRNA pairs. Functional and pathway enrichment analyses were predicted using a circRNA-miRNA-mRNA network.

**Results:**

RNA expression patterns were significantly different between normal and tumor samples. A total of 190 circRNAs, 76 miRNAs, and 678 mRNAs were differentially expressed. The analysis of the circRNA-miRNA-mRNA regulatory network revealed interactions between hsa-circ-0100153, hsa-miR-31, hsa-miR-767-3p, and hsa-miR-935 with *WASF3* in cancer. These interactions primarily function in DNA replication and the cell cycle.

**Conclusions:**

This study reveals a mechanism by which *WASF3* overexpression affects the expression of circRNAs hsa-circ-0100153, promoting BC progression by sponging hsa-miR-31/hsa-miR-767-3p /hsa-miR-935. This mechanism may increase the invasive potential of cancers, in addition to other reported molecular mechanisms involving the *WASF3* gene.

## Graphical abstract

**Figure:**
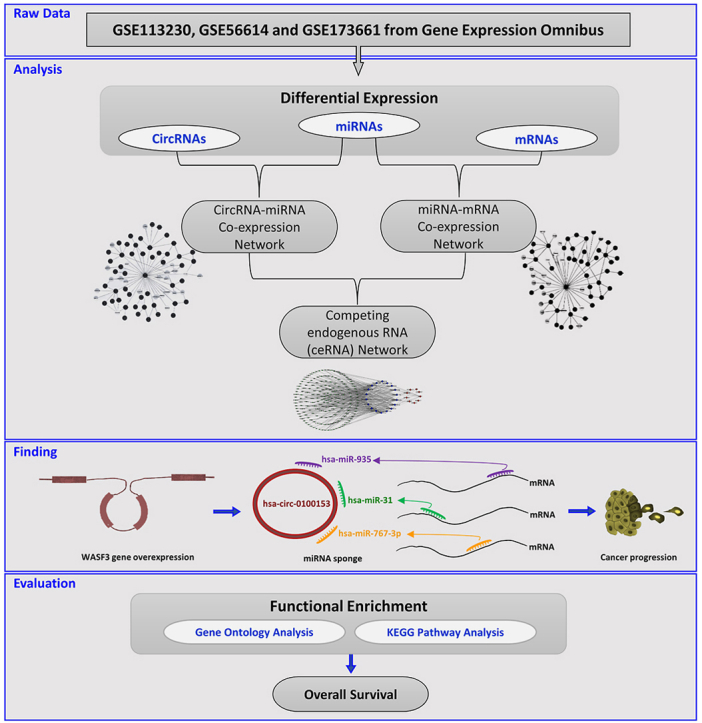

## 1 Introduction

Cancer is a devastating disease that remains one of the leading causes of death globally, with an estimated 10 million deaths each year [1]. Among the various types of cancer, breast cancer (BC) is the most commonly diagnosed form in women. Unfortunately, the primary cause of death in BC patients is often due to metastasis, which is the spread of cancer cells from the primary tumor to other parts of the body [2]. An essential gene identified for its pivotal role in promoting invasion and metastasis is *WASF3*, also known as *WAVE3*. This gene is a member of the Wiskott-Aldridge Syndrome (WAS) family of genes, which code for proteins that contain several highly conserved motifs [3]. These motifs include a WASP-homology-2 domain (WHD/V), a cofilin-homology domain (C), and an acidic (A) domain, all located at the C-terminal end of the protein. The verprolin central acidic (VCA) domain is particularly important because it coordinates the recruitment of monomeric actin and the ARP2/3 complex of proteins, which facilitate actin polymerization [4,5]. Actin polymerization is essential for cell movement and invasion, which are key processes in the development of metastatic cancer [6,7]. Research has shown that *WASF3* forms a complex with several other proteins, including the p85 component of PI3K and ABL kinase [8,9]. Additionally, other members of the *WASF* family, such as Abi1/2, CYFIP1/2, NCKAP1, and HSPC300, keep the protein in an inactive state. When activated, the protein complex is released, and the VCA domain becomes exposed, allowing the ARP2/3 complexes to bind and initiate actin polymerization, facilitating cell movement and metastasis [[10], [11], [12], [13]]. Thanks to previous research [3,11], our understanding of the function and role of *WASF3* in promoting metastasis has greatly improved. By identifying the complex interplay between various biological molecules involved in this process, we can develop more effective strategies to target this pathway and ultimately improve outcomes for cancer patients.

Recent studies have identified a new class of non-coding RNAs, called covalently closed circular RNAs (circRNAs), which can evade exonuclease-mediated degradation and are more stable in blood or plasma than linear RNAs [14,15]. As a result, circRNAs are considered ideal candidates for developing new diagnostic or prognostic biomarkers for cancers like BC [16,17]. CircRNAs have been shown to play a role in various BC hallmarks, including proliferation, apoptosis, and activating invasion and metastasis [18,19].

miRNAs are another type of non-coding RNA that regulates gene expression at the post-transcriptional level. CircRNAs have recently been discovered to regulate miRNA function by sponging miRNAs, which can either increase or decrease the expression levels of miRNAs [20,21]. For example, the oncogenic circRNA has-circ-0052112 enhances tumor cell invasion and migration by sponging miR-125a-5p, a tumor suppressor that inhibits the BAP1 oncogene. MiRNAs play essential roles in tumorigenesis, cancer invasion, metastasis, relapse, and drug resistance, making them attractive targets for cancer research [22].

**Fig. 1:**
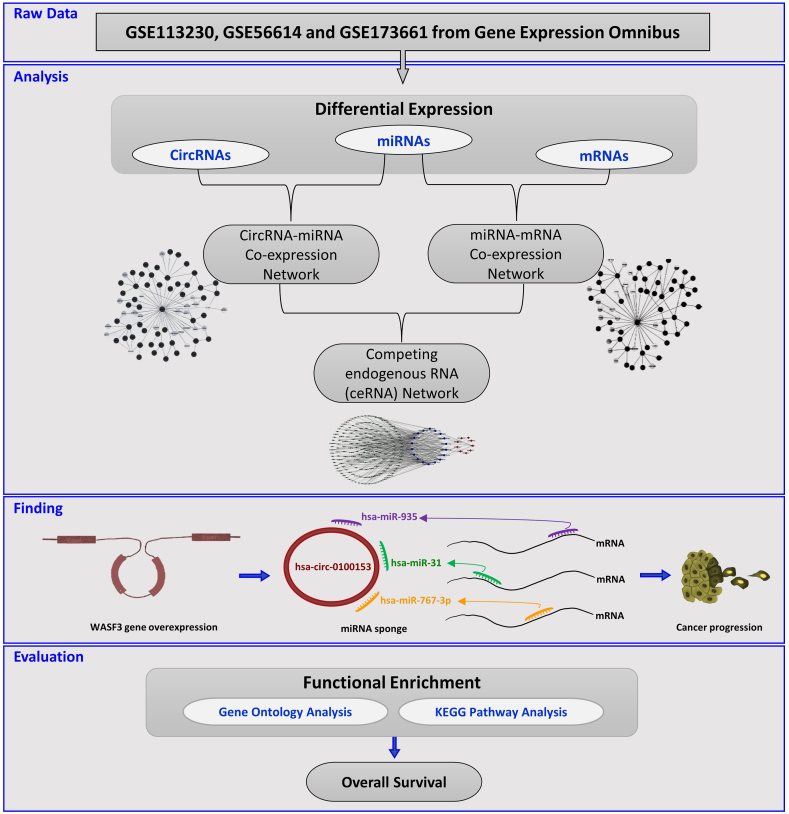
Graphical Abstract. The flowchart illustrates the study procedure.

In this study, we used a bioinformatics approach to identify additional functional pathways of the *WASF3* gene and highlight its role in the regulation of circRNAs and miRNAs in cancer progression. We collected expression profiles of circRNAs, miRNAs, and mRNAs in BC and normal breast tissues from the Gene Expression Omnibus (GEO) datasets and The Cancer Genome Atlas (TCGA) database. We identified differentially expressed mRNAs, circRNAs, and miRNAs using R software and reconstructed a circRNA-miRNA-mRNA regulatory network. We then predicted miRNA sponging by circRNA and miRNA target genes and assessed the competitive endogenous RNA (ceRNA) network using gene ontology (GO) annotation and Kyoto Encyclopedia of Genes and Genomes (KEGG) pathway analyses to determine the main functional pathways of BC. Graphical abstract (Fig. 1) shows the flowchart of the study procedure.

The results of this study provide insight into the functional pathways regulated by the *WASF3* gene and its role in BC progression. Additionally, the identification of circRNA-miRNA-mRNA regulatory networks and the ceRNA network can facilitate the development of new diagnostic and therapeutic strategies for BC treatment. This study contributes to our understanding of the genetic and epigenetic changes that drive BC progression and provides a foundation for further research in this field.

## 2 Materials and Methods

### 2.1 Raw data

In this research, to concurrently investigate the regulatory interactions between circRNA, miRNA, and mRNA, we chose and utilized three datasets from the GEO database (GSE113230, GSE56614, and GSE173661). These datasets comprised 100 samples of breast cancer and normal breast tissue, all processed through Illumina HiSeq 2000, 3000, 4000, and 5000 systems. To ensure data consistency and data uniformity, 47 samples from the GSEs were excluded due to factors such as drug interference or sequencing system incompatibility. Consequently, 49 breast cancer tissue samples and four normal breast tissue samples were retained for the study, resulting in a total of 53 samples for analysis. By leveraging these valuable resources in the GEO database, researchers can gain insights into the molecular underpinnings of breast cancer and ultimately improve diagnosis and treatment options for patients.

### 2.2 Data pre-processing

Trimmomatic-0.4 was employed to filter the raw data obtained from RNA sequencing by removing adapter sequences, low-quality reads, and invalid reads [23,24]. The resulting clean reads were further processed to eliminate any rRNA residues by comparing against known rRNA information in the RNA central database. Subsequently, these clean reads were aligned to the human reference genome-HG38 available in the UCSC genome database. External factors such as sample preparation or sequencing process that are not biologically relevant may affect the expression of individual samples, and as such, it is necessary to ensure that all samples have a similar range and distribution of expression values. Normalization is required to achieve this, and the R software's “edgeR” package (version 3.50.3) was used for normalization of raw data and subsequent data processing. The package ensures that the expression distributions of each sample are consistent throughout the experiment. The ability to accurately normalize RNA sequencing data is crucial for reliable downstream analysis and ultimately leads to a better understanding of gene expression in various biological contexts [25].

### 2.3 Analysis of mRNAs differential expression (DE)

To preprocess the RNA sequencing data, Trimmomatic-0.36 was utilized to filter out low-quality reads, adapter sequences, and undetermined bases. Next, rRNA residues were removed to ensure high-quality clean reads. The clean reads were aligned to the reference human genome-HG38 using TopHat 2.1.1 version, with annotation references selected from the RefSeq databases. The mRNA expression was calculated using the Reads Per Kilobase per Million of reads method. The limma package in R software was used to calculate the adjusted P-value (adj. p) and the absolute log value of fold-change (log|FC|). The selection of differentially expressed mRNAs (DE-mRNAs) was based on the criteria of adj. p < 0.05 and log|FC| > 1. This approach allowed for the identification of transcripts that showed significant differences in expression between experimental conditions, thereby providing insights into the molecular mechanisms underlying breast cancer [26,27].

### 2.4 Analysis of CircRNA differential expression

The TopHat-Fusion algorithm was utilized to detect candidate circRNAs from the unmapped reads. The algorithm applied several criteria, including back-spliced junction reads greater than one, a maximum of two mismatches, and the presence of canonical splicing signals (GT/AG) between the two splice sites, with a maximum distance of 100 kb. The circRNA expression was quantified as reads per million mapped reads [28]. Differential expression analysis of circRNAs was performed using the “edgeR” package of R software, and the selection criteria were based on the adjusted p-value less than 0.05 and a log|FC| greater than one. These criteria were chosen to ensure the selection of circRNAs with significant expression changes between different experimental conditions.

### 2.5 Analysis of miRNA differential expression

The raw reads obtained from the miRNA library were processed using Cutadapt version 3.7 to remove adapter sequences and low-quality reads. BWA version 0.7.17 was used to map the clean reads to the HG38 reference genome to identify miRNAs. Known miRNAs were downloaded from the miRBase database version 22 and used to identify each group's miRNAs. The miRNA expression was calculated using the reads per million clean tags (RPM) method. The identification of differentially expressed (DE) miRNAs was based on log|FC| >1 and an adjusted p-value (adj. p) of less than 0.05, indicating statistical significance. These criteria were employed using R software's “edgeR” package for data analysis.

### 2.6 Prediction of miRNA binding sites in circRNA

The Circular RNA Interactome (CircInteractome) [29] and Cancer-Specific CircRNA (CSCD) [30] were used to predict miRNA binding sites. The interactions of miRNAs in these two databases were considered as potential target miRNAs for the DE circRNAs. DE miRNAs were then further screened for these potential target miRNAs based on The Cancer Genome Atlas (TCGA) data. The miRNAs that were found to be significantly dysregulated in TCGA and inversely correlated with the expression of the circRNA were selected as potential miRNA regulators of the circRNA [31,31].

### 2.7 CircRNA-miRNA Co-expression network analysis

To identify miRNAs that potentially target DE circRNAs, we employed three miRNA target prediction tools - Miranda, TargetScan, and RNAhybrid. We set the following thresholds to pick out candidate miRNA-circRNA relationships: Total Score ≥140 and Total Energy < −17 k mol for Miranda, minimum free energy ≤ −21 for RNAhybrid, and conserved groups for TargetScan. The potential miRNA-circRNA interactions were then identified using these criteria [32].

### 2.8 miRNA-mRNA Co-expression network analysis

To identify potential target DE mRNAs of the circRNA-miRNA network, several databases such as MiRDB, MiRTarBase, and MiRWalk were used for miRNA target prediction. The intersection of mRNAs predicted by at least two of these databases was considered to create a reliable miRNA-mRNA network [33].

### 2.9 Competing endogenous RNA (ceRNA) network analysis

To construct the circRNA-miRNA-mRNA regulatory network, we utilized a combination of circRNA-miRNA pairs and miRNA-mRNA pairs. Our dataset comprised only differentially expressed (DE) circRNAs, miRNAs, and mRNAs which can be accessed on the CSCD, CircInteractome, and CircBase databases. We assessed the co-expression relationship between circRNAs, miRNAs, and mRNAs using the Pearson correlation coefficient (r) and correlation P-value (CP-value). The interactions with a strong correlation threshold of r > 0.85 or r < −0.85 and a CP-value <0.05 were selected to establish the ceRNA network. The Cytoscape 3.9.1 software was used to visualize the ceRNA network [34,35].

### 2.10 GO and KEGG functional enrichment analysis

To gain deeper insights into the mechanisms, we conducted Kyoto Encyclopedia of Genes and Genomes (KEGG) pathway analysis and Gene Ontology (GO) enrichment analysis to assess the advanced functions of differentially expressed (DE) mRNA, circRNAs, and miRNA. KEGG analysis provides annotation information on signal transduction and biological systems based on large-scale molecular data generated by high-throughput experimental techniques. GO annotates genes and categorizes them based on molecular functions and cellular components. To determine the function of DE genes in KEGG pathways, we employed the KOBAS 3.0 software. A statistically significant difference was determined based on an adjusted p-value of <0.05 [[36], [37], [38]].

### 2.11 Survival analysis

We obtained Breast cancer mRNA expression profile data and clinical sample data from the TCGA database for survival analysis of mRNAs, circRNAs, and miRNAs selected from the ceRNA network. We employed Kaplan-Meier curves for overall survival analysis and used the log-rank test for statistical analysis. A P-value less than 0.05 was considered as the cutoff point for prognostic survival significance. This enabled us to determine the association between gene expression and patient survival, providing insights into the prognostic significance of the ceRNA network in breast cancer [39].

## 3 Results

### 3.1 Differentially expressed circRNAs, miRNAs, and mRNAs

**Fig. 2:**
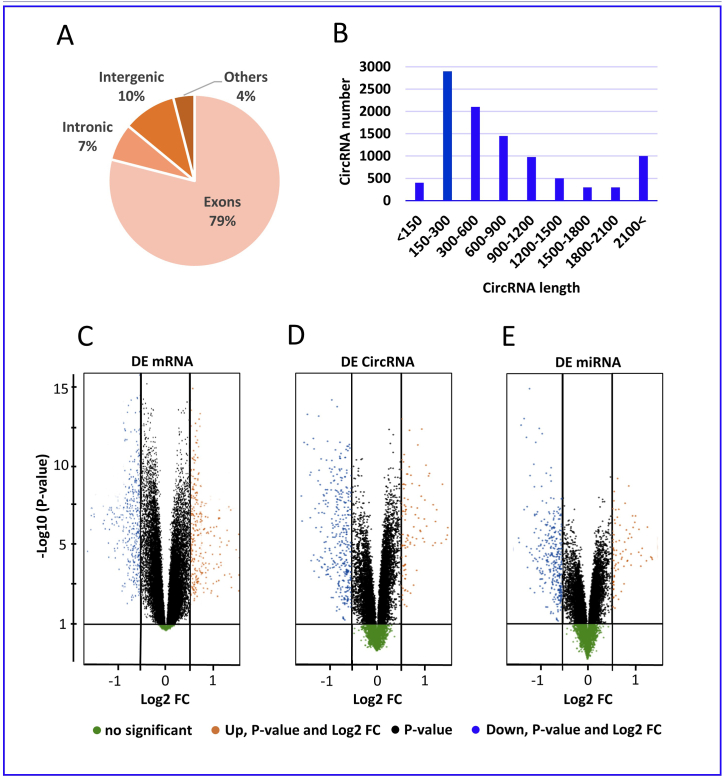
The distribution and regulation of different RNAs. (A) The pie chart shows the distribution of circular RNAs (circRNAs) based on their location within the genome. The chart indicates that 79 % of circRNAs are composed of exons, 10 % map to intronic regions, and 7 % are located in intergenic regions. (B) Chart shows the size distribution of circular RNAs, with 80 % having a predicted spliced length of less than 800 nucleotides. The volcano plots in C, D, and E demonstrate that circRNAs, miRNAs, and mRNAs were differentially regulated between breast cancer and normal samples.

In this study, we utilized clean reads to identify circular RNAs (circRNAs) and found that the majority of these reads were mapped to the reference genome. Specifically, we discovered that 79 % of circRNAs are composed of exons, 10 % map to intronic regions, and 7 % are located in intergenic regions (as shown in Fig. 2A). Our analysis also revealed that circRNAs vary in size, with a range of 150–2500 nucleotides. Notably, 80 % of circRNAs have a predicted spliced length of less than 800 nucleotides, 50 % have a length of less than 500 nucleotides, and 25 % have a length between 500 and 1000 nucleotides (as shown in Fig. 2B).

In our study, we compared the expression profiles of mRNAs in breast cancer and normal samples and found 678 differentially expressed mRNAs, with 586 being upregulated and 92 being downregulated. The majority of these DE mRNAs were found to be significantly upregulated (as shown in Fig. 2C).

Additionally, using the filtering method described in Materials and Methods, we identified 190 DE circRNAs between the breast cancer and normal samples, with 77 being upregulated and 113 being downregulated (as shown in Fig. 2D). These DE circRNAs were distributed across multiple chromosomes, with many being upregulated on chromosomes 1, 2, 3, 9, 11, 12, 17, 19, 20, 21 and 22. Thirteen down-regulated circRNAs were found on various chromosomes.

Using sequencing analysis, we also investigated miRNA expression profiles in breast cancer samples and found that 76 miRNAs were significantly dysregulated, with 29 being upregulated and 47 being downregulated (as shown in Fig. 2E).

**Fig. 3:**
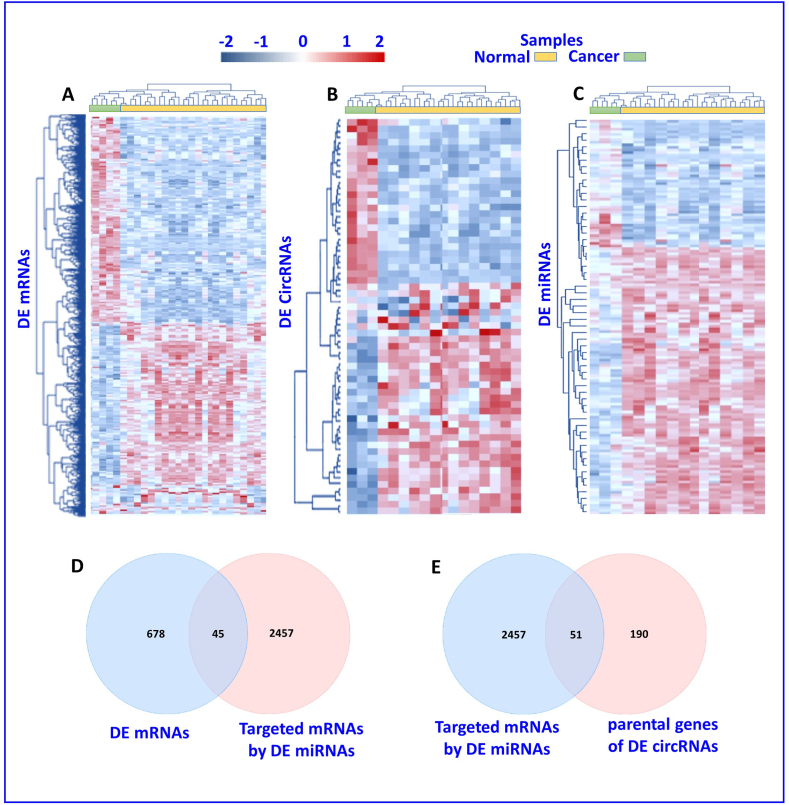
Differential Expression Analysis of circRNA, miRNA, and mRNA. A-C shows the hierarchical clustering analysis of differentially expressed circRNA, miRNA, and mRNA. Dysregulated miRNAs were mostly upregulated. The analysis clearly distinguishes breast cancer and normal groups. D and E depict Venn diagrams indicating 45 joint mRNAs between DE mRNAs and miRNA targets, and 51 joint mRNAs between miRNA targets and parental genes of DE circRNAs.

Our analysis identified 2457 candidate genes as target genes for the top 38 DE miRNAs. Additionally, we applied hierarchical cluster analysis to all DE circRNA, miRNA, and mRNA and the results are illustrated in Fig. 3 (A-C). The majority of dysregulated circRNAs were found to be upregulated and the hierarchical clustering analysis clearly revealed the difference between the breast cancer and normal groups. The Venn diagrams in Fig. 3 D and **E** show that there are 45 joint mRNAs between DE mRNAs and mRNAs targeted by DE miRNAs and 51 joint mRNAs between mRNAs targeted by DE miRNAs and parental genes of DE circRNAs.

### 3.2 Construction of the circRNA, miRNA, and mRNA ceRNA networks

**Fig. 4:**
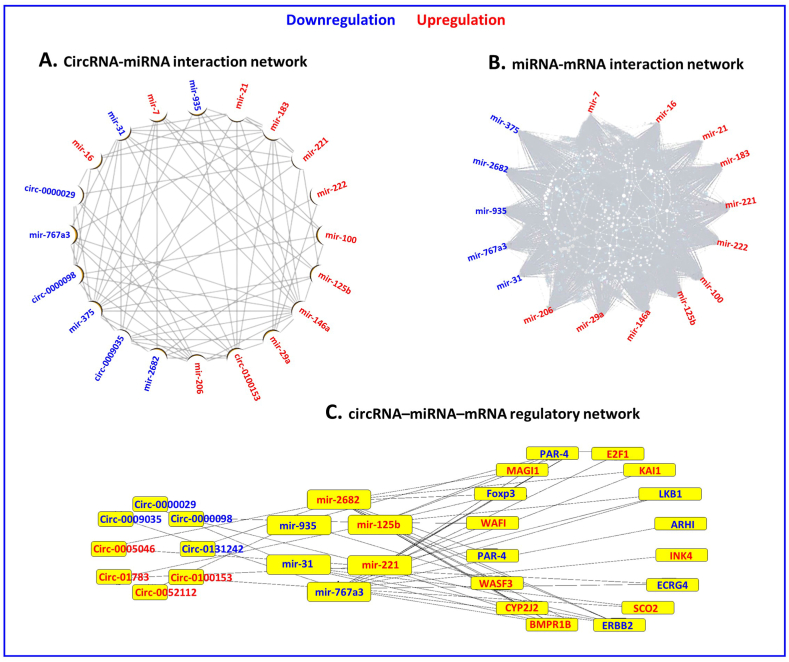
Network Analysis of Circular RNAs, miRNAs, and mRNAs (A) shows the CircRNA-miRNA network analysis results, revealing 16 miRNAs with potential interactions with eight circRNAs. (B) Depicts a miRNA-mRNA interaction network created by predicting 320 targets for each of the 16 DE miRNAs. (C) represents a ceRNA network established by integrating circRNA-miRNA and miRNA-mRNA interactions, identifying important nodes in breast cancer.

Recent studies indicate that circRNAs may indirectly regulate gene expression by binding to miRNAs [[40], [41], [42]]. To understand how DE circRNAs are involved in this process, a comprehensive analysis of the interactions between circRNAs and miRNAs was conducted. Results of the CircRNA-miRNA network analysis revealed 16 miRNAs that had potential interactions with eight circRNAs, with eleven miRNAs being up-regulated and five miRNAs being down-regulated (as shown in Fig. 4A). A miRNA-mRNA interaction network was then created by predicting 320 targets for each of the 16 DE miRNAs (shown in Fig. 4B). Finally, by integrating the circRNA-miRNA and miRNA-mRNA interaction networks and by adding the top ranks of three DE groups, a ceRNA network was established to investigate the role of circRNAs in breast cancer. This analysis identified 8 circRNAs, 6 miRNAs, and 16 mRNAs that may serve as important nodes in this ceRNA network (shown in Fig. 4C).

### 3.3 GO and KEGG pathway enrichment analysis

**Fig. 5:**
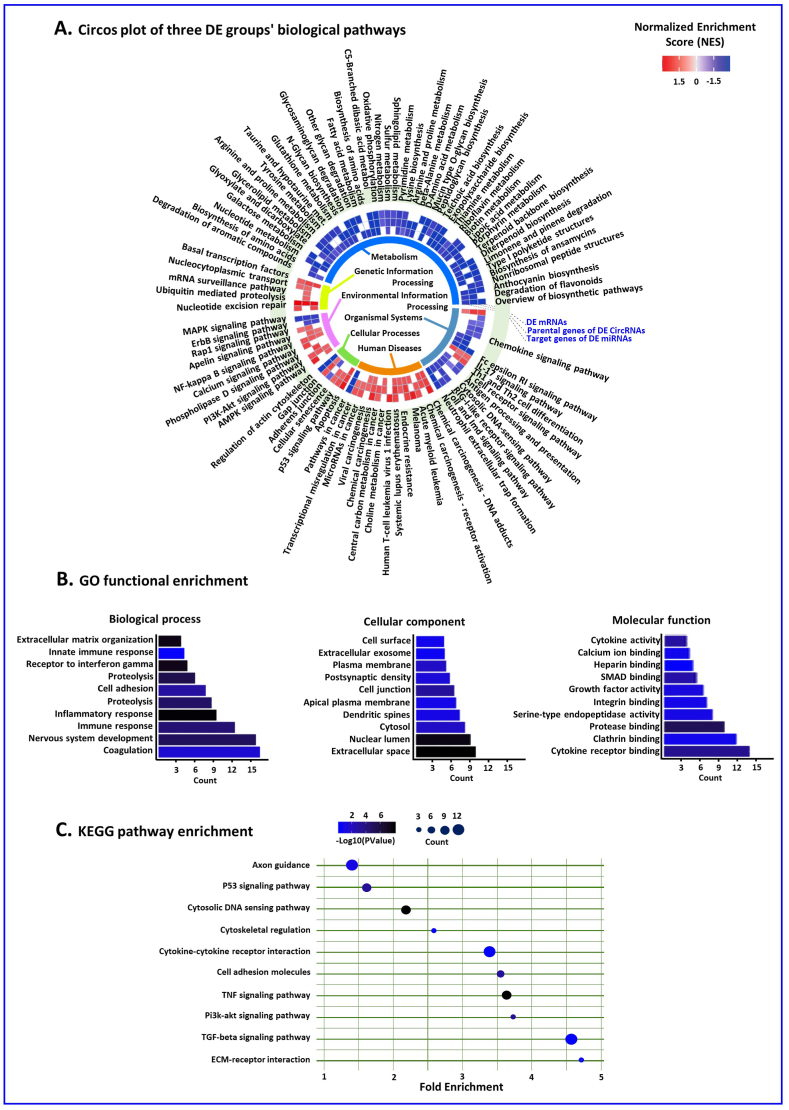
Pathway Analysis of Circular RNAs, miRNAs, and mRNAs. (A) The circus plot shows cross-dataset pathway results of differentially expressed mRNAs, circRNAs, and miRNAs, indicating metabolic pathways' significant role in breast cancer. (B) Gene ontology analysis of extracted mRNAs, parental genes of circRNAs, and target genes of miRNAs from the ceRNA network. Enrichment in cancer-related biological processes such as “extracellular matrix organization” and “growth factor activity”. (C) Kyoto Encyclopedia of Genes and Genomes pathway analysis of extracted mRNAs, parental genes of circRNAs, and target genes of miRNAs from the ceRNA network. Enrichment in cancer-related pathways such as “p53 signaling pathway” and “ECM-receptor interaction."

The cross-dataset pathway results of differentially expressed mRNAs, circRNAs, and miRNAs in Fig. 5A's circus plot suggest that metabolic pathways play a significant role in breast cancer. As shown in Fig. 5B and **C**, we performed gene ontology (GO) and Kyoto Encyclopedia of Genes and Genomes (KEGG) pathway analyses on extracted mRNAs, parental genes of circRNAs, and target genes of miRNAs from the competing endogenous RNA network. Our analysis revealed a significant enrichment of cancer-related GO items and KEGG pathways. For example, GO annotation analysis identified biological processes such as “extracellular matrix organization,” “receptor to interferon gamma,” “extracellular space,” and “growth factor activity” (Fig. 5B). Additionally, KEGG pathway enrichment analysis showed enrichment in pathways such as “p53 signaling pathway,” “cytosolic DNA sensing pathway,” “cell adhesion molecules,” and “ECM-receptor interaction” (Fig. 5C).

## 4 The identification of circRNA-miRNA interactions

**Fig. 6:**
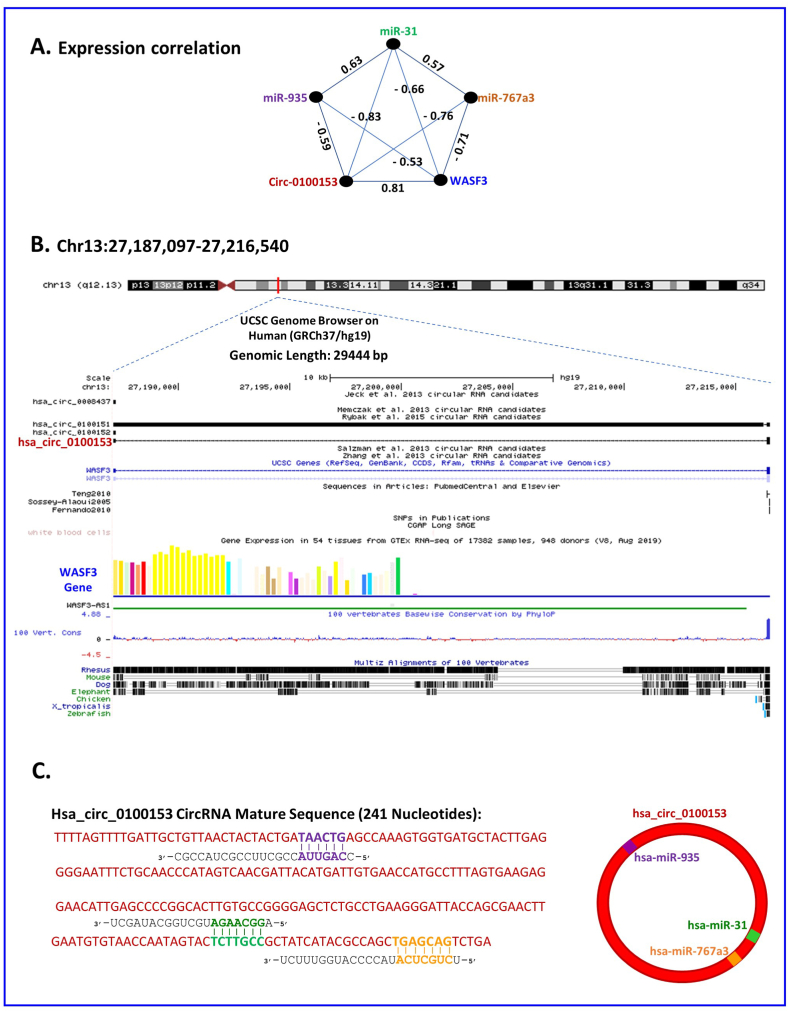
Correlation and Binding Site Analysis of Circular RNAs, miRNAs, and mRNAs. (A) plot shows a positive correlation between the expression of WASF3 and the circular RNA hsa-circ-0100153, as well as a negative correlation between these two and three miRNAs (hsa-miR-31, hsa-miR-767-3p, and hsa-miR-935). (B) Shows the parental gene of hsa-circ-0100153, WASF3, identified on chromosome 13 using the UCSC Genome Browser. (C) Shows predicted miRNA binding sites for hsa-miR-31, hsa-miR-767-3p, and hsa-miR-935 on the circular RNA hsa-circ-0100153 using web tools, Circular RNA Interactome (circBase), CSCD, and Circbank.

In Fig. 6A, we observed a positive correlation between the expression of *WASF3* and the circular RNA hsa-circ-0100153, as well as a negative correlation between these two and three miRNAs (hsa-miR-31, hsa-miR-767-3p, and hsa-miR-935). Using the UCSC Genome Browser, we identified the parental gene of hsa-circ-0100153, *WASF3*, on chromosome 13 (Fig. 6B). To further investigate the structural patterns of the circRNA and predict miRNA binding sites, we used the Circular RNA Interactome (circBase) and two web tools, CSCD and Circbank. As a result, we predicted miRNA binding sites for hsa-miR-31, hsa-miR-767-3p, and hsa-miR-935 on the circular RNA hsa-circ-0100153 (Fig. 6C).

## 5 Determination of prognostic markers

**Fig. 7:**
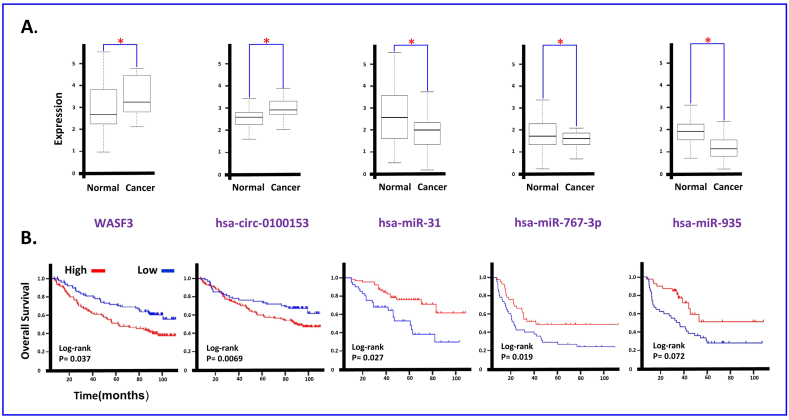
RNA Expression and Survival Analysis in Breast Cancer (A) shows the expression differences between cancer and normal tissues of different RNAs and WASF3 in breast cancer. (B) Shows the Kaplan-Meier survival curves that demonstrate the relationship between the expression.

Fig. 7A shows the difference in expression between cancer and normal in different RNAs and *WASF3*. The Kaplan-Meier method was used to calculate survival curves and investigate the relationship between expression patterns and clinical information. The results revealed that high expression of *WASF3* and circular RNA hsa-circ-0100153 correlated with shorter overall survival (Log-rank, P < 0.05). Additionally, low expression of hsa-miR-31, hsa-miR-767-3p, and hsa-miR-935 were associated with shorter overall survival (Log-rank, P < 0.05). These findings are illustrated in Fig. 7B.

## 6 Discussion

The *WASF3* gene is a key player in the *WASF* gene family which plays a critical role in regulating the actin cytoskeleton [43]. This gene has been found to be commonly overexpressed in several types of cancer, including breast, lung, and ovarian cancer. The overexpression of the *WASF3* gene has been linked to several cancer-promoting activities, including increased cell proliferation and migration, as well as resistance to chemotherapy [44]. Some studies suggest that *WASF3* may regulate the activity of other genes involved in cell growth and division, or interact with proteins that are important for cancer development [45]. Despite the growing body of evidence pointing to the importance of the *WASF3* gene in cancer, the exact mechanism by which it contributes to cancer development and progression is still not completely understood. More research is needed to gain a better understanding of the *WASF3* gene's role in cancer and to develop targeted therapies that can effectively inhibit its activity in cancer cells. The results of present research using bioinformatics tools suggest the existence of a regulatory mechanism between the *WASF3* gene, circular RNA hsa-circ-0100153, and miRNAs (hsa-miR-31, hsa-miR-767-3p, and hsa-miR-935) that appears to affect the intensity of cancer invasion. Although the influence of this mechanism on cancer invasion cannot be fully assessed in the current study, it remains a promising therapeutic target for further researches.

The study of circular RNA hsa-circ-0100153 has shown a positive correlation between its expression and that of the *WASF3* gene (as shown in Fig. 6A). This indicates that an increase or decrease in the expression of the *WASF3* gene leads to a coordinated increase or decrease in the expression of circular RNA hsa-circ-0100153. *WASF3* is a gene that encodes for the Wiskott-Aldrich syndrome protein family member 3, which is known to play a role in cancer development and progression [45]. The results of gene expression analysis in healthy and cancer patients, as well as the survival analysis, show that an increase in the expression of the *WASF3* gene is visible in cancer patients, leading to lower survival rates (as shown in Fig. 7A and B). This is because *WASF3* regulates actin cytoskeleton organization and cell migration, which are essential for tumor invasion and metastasis, and its overexpression has been linked to poor prognosis in various types of cancer, including breast, lung, and gastric cancer [4]. On the other hand, this study have shown that hsa-circ-0100153 is upregulated in cancer (as shown in Fig. 7A) and may play a role in cancer progression by regulating the expression of *WASF3* protein [46]. The gene expression analysis of healthy individuals and cancer patients, along with the survival analysis, show that cancer patients have higher expression of circular RNA hsa-circ-0100153 (as shown in Fig. 7A and **B**), which may explain their lower survival rates [47]. The positive correlation between the expression of *WASF3* and hsa-circ-0100153 (as shown in Fig. 6A) suggests that the increase in *WASF3* expression through back-splicing causes an increase in the synthesis and expression of circular RNA [48] hsa-circ-0100153 (as shown in Fig. 6B). The fact that circular RNA hsa-circ-0100153 also regulates the expression of *WASF3* cannot be evaluated from the data, but the increase in the expression of both has been associated with lower patient survival. In conclusion, hsa-circ-0100153 has been found to be a potential biomarker for cancer diagnosis and prognosis, and *WASF3* has been identified as a potential therapeutic target for cancer treatment [43]. These findings may pave the way for further research and development of new cancer therapies and diagnostics.

Circular RNA and its relationship with miRNAs have been widely researched. miRNAs are small, non-coding RNA molecules that control gene expression by binding to target messenger RNAs (mRNAs), leading to degradation or repression of translation [49,50]. Our study found that hsa-miR-31 and hsa-circ-0100153 expression are negatively correlated, meaning an increase in one results in a decrease in the other. This connection is further shown in Fig. 6, where hsa-circ-0100153 is seen to sponge hsa-miR-31 [51,52], thus creating a negative correlation. Studies have shown hsa-miR-31's role in the development of cancer, including breast, lung, and prostate cancer [53]. Results from our analysis of healthy and cancer patients' gene expression and survival analysis in Fig. 7 show a reduced hsa-miR-31 expression in cancer patients, contributing to their short survival rate. hsa-miR-31 also regulates cancer cell proliferation, migration, invasion, and angiogenesis through pathways such as Wnt/beta-catenin, TGF-beta, and PI3K/Akt [54,55].

Similarly, hsa-miR-935 is a miRNA implicated in the development of cancer, including liver, lung, and gastric cancer [42]. Our gene expression studies and survival analysis in Fig. 7 reveal a reduced hsa-miR-935 expression in cancer patients. Our study found a negative correlation between hsa-miR-935 expression and hsa-circ-0100153 expression, with an increase in one leading to a decrease in the other. This connection is further demonstrated in Fig. 6, where hsa-circ-0100153 is seen to sponge hsa-miR-935, thus creating a negative correlation [49,50,52].

Similarly, hsa-miR-767-3p and hsa-circ-0100153 expression are negatively correlated, with an increase in one resulting in a decrease in the other. Fig. 6 also illustrates hsa-circ-0100153's connection band to hsa-miR-767-3p, indicating that hsa-circ-0100153 sponging hsa-miR-767-3p creates a negative expression correlation. hsa-miR-767-3p′s alterations in expression have been observed in various cancers, including breast, lung, and colorectal cancer [41]. Our gene expression studies and survival analysis in Fig. 7 show a reduced hsa-miR-767-3p expression in cancer patients, which may contribute to their short survival rate. hsa-miR-767-3p regulates cell proliferation, migration, invasion, and angiogenesis and plays a role in tumorigenesis pathways such as the PI3K/Akt and ERK pathways [56]. Fig. 4 also displays related biological pathways based on our analysis of gene ontology (GO) terms and Kyoto Encyclopedia of Genes and Genomes (KEGG) pathways.

Despite the fact that, the relationship between *WASF3* and hsa-circ-0100153, hsa-miR-31, hsa-miR-767-3p, and hsa-miR-935 appears to be complex and multifaceted, the interaction between hsa-circ-0100153 and these miRNAs can modulate gene expression and play a role in the regulation of various biological processes. The relationship between hsa-circ-0100153 and miRNAs (hsa-miR-31, hsa-miR-767-3p, hsa-miR-935) is complex and affects gene expression. The impact of *WASF3* gene on hsa-circ-0100153 further complicates these interactions and requires further study. In this study, we evaluated the effect of *WASF3* on hsa-circ-0100153 but couldn't identify the reverse effect due to the network's undirected nature. We also identified hsa-circ-0100153's sponge effect on the miRNAs, but couldn't evaluate their impact on hsa-circ-0100153 or *WASF3* due to data limitations.

## 7 Conclusion

In conclusion, our study elucidates the impact of *WASF3* overexpression on breast cancer progression through intricate regulatory networks. We confirmed *WASF3*'s influence on hsa-circ-0100153 and identified its miRNA sponge effect on hsa-miR-31, hsa-miR-767-3p, and hsa-miR-935. Despite challenges in discerning the reverse effects due to network complexity and data limitations, our findings shed light on *WASF3*-mediated circRNA-miRNA interactions. This study underscores the potential of targeting this mechanism for therapeutic interventions, providing a foundation for further in-depth investigations into cancer invasion dynamics.

## Ethical approval

Not applicable.

## Consent for publication

Not applicable.

## Availability of data and materials

A majority of the data generated or analyzed during this study are included in this published article. The datasets generated and/or analyzed during this study can be obtained from the corresponding author upon reasonable request.

## Funding

The author(s) received no financial support for the research, authorship, and/or publication of this article.

## CRediT authorship contribution statement

**Majid Mokhtari:** Conceptualization, Data curation, Formal analysis, Funding acquisition, Investigation, Methodology, Project administration, Resources, Software, Validation, Visualization, Writing - original draft, Writing - review & editing. **Samane Khoshbakht:** Conceptualization, Data curation, Formal analysis, Funding acquisition, Investigation, Methodology, Writing - review & editing. **Mohammad Esmaeil Akbari:** Supervision. **Moravveji Sayyed Sajjad:** Writing - review & editing.

## Declaration of competing interest

The authors declare that they have no known competing financial interests or personal relationships that could have appeared to influence the work reported in this paper.
